# Anal Sphincter Defect and Fecal Incontinence

**DOI:** 10.1097/PG9.0000000000000254

**Published:** 2022-10-20

**Authors:** Sherief Mansi, Karla Vaz, Neha R. Santucci, Khalil El-Chammas, Kahleb Graham, Nelson G. Rosen, Ajay Kaul

**Affiliations:** From the * Department of Pediatrics, Division of Gastroenterology, Cincinnati Children’s Hospital Medical Center (CCHMC), University of Cincinnati, Cincinnati, OH; † Department of Pediatrics, Division of Gastroenterology, Hepatology and Nutrition, Nationwide Children’s Hospital, The Ohio State University, Columbus, OH; ‡ Division of Pediatric General and Thoracic Surgery, Cincinnati Children’s Hospital Medical Center (CCHMC), Cincinnati, OH.

## Abstract

Anal sphincter defects can lead to fecal incontinence. The relationship between the extent of defect and continence is controversial. Magnetic resonance imaging (MRI) of the pelvis can assess anal sphincter defects. Transrectal ultrasonography (TRUS) is used to assess sphincter integrity in adults. We present a 17-year-old male with history of sexual abuse, rectal prolapse, and fecal incontinence. MRI showed a small defect that did not explain his clinical presentation. TRUS identified more extensive defects which were not picked up by MRI. The patient had rectopexy, and his rectal prolapse and fecal incontinence resolved. TRUS was superior in identifying sphincter defects compared with MRI. Our case also highlights that continence is possible despite large sphincter defects in pediatric patients. This may reflect the compensatory mechanism of residual sphincter in the absence of the aggravating factors like rectal prolapse.

## INTRODUCTION

Anal sphincter defects can lead to fecal incontinence in patients with anorectal malformations and complications after surgery for Hirschsprung’s disease. Contrast enemas and magnetic resonance imaging (MRI) are commonly used to evaluate anorectal anatomy in these cases. Transrectal ultrasonography (TRUS) is used as a diagnostic tool in adults in disorders including anorectal tumors, prostate cancer, anorectal disease in patients with inflammatory bowel disease (IBD) and anal sphincter evaluation in child-bearing females with postpartum fecal incontinence (1, 2). Combined with other modalities such as anorectal manometry and pudendal nerve latency, TRUS helps correlate ultrasonography findings with sphincter function (3).

Recently, TRUS has been used in some pediatric centers as an adjunctive noninvasive diagnostic tool in anorectal disease related to IBD and anal sphincter evaluation in children with fecal incontinence and traumatic injury (4). However, no reports have been published on the relationship between the degree of anal sphincter defect and incontinence in pediatric patients.

## CASE REPORT

A 17-year-old Caucasian adopted male with a history of autism spectrum disorder and functional constipation presented for evaluation of rectal prolapse, fecal incontinence, and hematochezia. Adoptive mom, the current care giver, noted a 3–4 cm rectal prolapse requiring reduction, with and without straining, rectal bleeding, perineal discomfort, and a sensation of incomplete evacuation despite having daily, soft, stools on his laxative regimen. He has a history of physical and sexual abuse with anal penetration when he was living with his biological parents. Thyroid screening celiac serology, and sweat chloride test were normal. Defecography showed weakness of the pelvic floor with rectal prolapse and pelvic floor descent. The prolapse was confined to the rectum without sigmoid colon involvement. A water soluble contrast enema showed normal colonic caliber with redundancy. Upper and lower endoscopy were normal except for a small inflammatory rectal polyp that was removed.

**FIGURE 1.**
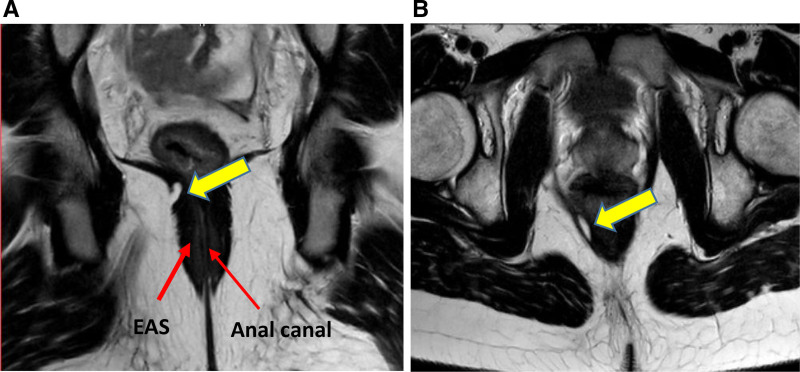
Pelvic MRI showing: (A) T2-coronal oblique and (B) T2-axial views. MRI = magnetic resonance imaging. The yellow arrows show the fat protrusion through the external anal sphincter (EAS) defect reported only at 8 o’clock (lithotomy position). The anal canal in collapsed in the midline (figure 1A) with no abnormality identified in the internal anal sphincter (IAS), (thin wall lining the anal canal).

**FIGURE 2.**
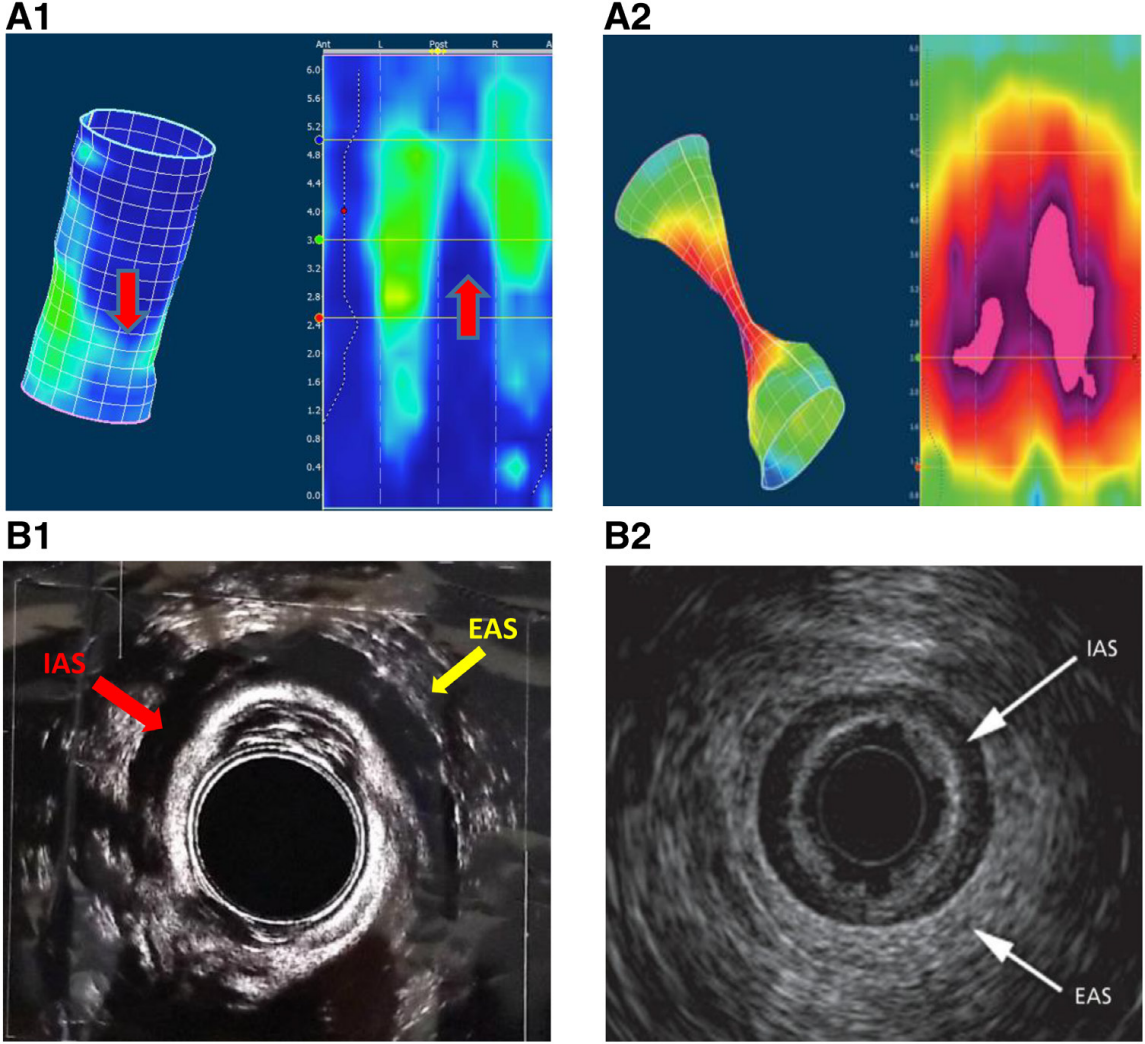
(A1) Shows 2D and 3D images from HRAM showing sphincter defects indicated by low pressures (red arrows). (A2) shows a normal 2D and 3D anorectal manometry5. (B1) TRUS shows both the intact parts of the IAS, (red arrow—inner hypoechoic ring) with defect extending from 3 to 9 o’clock and EAS, (yellow arrow—outer hyperechoic ring) with a defect from 5 to 8 o’clock. (B2) shows a normal TRUS image of normal sphincters. Normally the IAS and EAS are complete circles with no interruption. EAS = external anal sphincter; HRAM = high resolution anorectal manometry; IAS = internal anal sphincter; TRUS = transrectal ultrasonography.

Pelvic MRI showed a small defect in the external anal sphincter (EAS) at 8 o’clock (Fig. 1). Anorectal manometry (ARM) showed a low resting sphincter pressure (mean of 30 mmHg, normal 33–101 mm Hg), weak squeeze, normal rectoanal inhibitory reflex signifying intact enteric nervous system (with increased relaxation with balloon volume increase signifying intact spinal reflexes), and pelvic floor dyssynergia. 3D images of the ARM (Medtronic) were concerning for more extensive sphincter defects (Fig. 2A) (5). TRUS (BK3000) showed more extensive defects in both the EAS (from 5 to 8 o’clock) and internal anal sphincter (IAS) from 3 to 9 o’clock (Fig 2B) (6). The hematochezia resolved postpolypectomy with no change in medications; however, rectal prolapse persisted requiring rectopexy with sigmoid resection for redundancy likely contributing to the prolapse. No symptoms were reported at 12-month follow-up, and he has not needed surgical intervention to repair his sphincter defect.

## DISCUSSION

Previous studies compared the sensitivity and specificity of anal endoscopic ultrasonography (EUS) and MRI in identification of pelvic IBD where the EUS accuracy was comparable to MRI, though EUS is slightly different than TRUS (7–9). TRUS utilizes a rigid ultrasound probe with no endoscopy, which can be done in an office setting and is easier to train on by healthcare providers (10). TRUS is a less expensive diagnostic tool, usually tolerated well with no complications and can be done at bedside in cooperative patients.

In our patient, TRUS was superior to MRI in identifying both the site and the degree of anal sphincter defects. MRI identified a small tear in the EAS at 8 o’clock, inconsistent with the clinical history. The TRUS identified a larger defect involving almost one-third of the EAS. In addition, due to the small thickness of the IAS wall, a significant defect involving half of the IAS was missed by MRI and was only visible with TRUS. This may explain the persistent rectal prolapse in our patient despite adequate control of constipation. The TRUS anatomical findings were consistent with functional testing including ARM findings (11, 12) as well as defecography. While the TRUS result was not the only decisive factor, it helped make the decision to proceed with surgery, as conservative treatment was not successful with these extensive tears.

In conclusion, this case underscores the superiority of TRUS over MRI in better characterizing anal sphincter defects. Furthermore, this case indicates that continence is possible despite a significant sphincter defect. This reflects the compensatory mechanisms of the residual sphincter in the absence of other aggravating factors like rectal prolapse. Based on its accuracy, safety and cost, we suggest that TRUS be used more often for evaluation of fecal incontinence in children.

## ACKNOWLEDGMENTS

Informed signed consent was obtained from the patient’s parent (adoptive mother) to publish the details of this case.
